# Caregiver Abuse of Chicago Chinese Older Adults in a Community-Dwelling Population

**DOI:** 10.23937/2469-5858/1510004

**Published:** 2015-09-05

**Authors:** Xin Qi Dong, Ge Li

**Affiliations:** Rush Institute for Healthy Aging, Rush University Medical Center, USA

**Keywords:** Elder abuse, Adult children, Caregiver, Chinese

## Abstract

**Objectives:**

This study aimed to examine the prevalence and correlates of elder abuse reported by adult children among U.S Chinese populations.

**Method:**

A community-based participatory research approach was implemented. A total of 548 Chinese adult children aged 21 years and over participated in this study. Elder abuse reported by adult children was assessed using Caregiver Abuse Screen (CASE).

**Results:**

This study found a prevalence of 59.8%for elder abuse among 548 adult children. Younger age (r = −0.10, p < .05), higher level of education (r = 0.20, p < .001), higher income (r = 0.14, p < .01), more years in the U.S. (r = 0.12, p < .05), not born in Mainland China (r = −0.13, p < .01), and English-speaking (r = 0.16, p < .001) were positively correlated with elder abuse reported by adult children.

**Discussion:**

Elder abuse by adult children is prevalent among U.S. Chinese populations. It is necessary for researchers, health care providers and policy makers to put more attention on elder abuse by adult children. Longitudinal research is needed to explore the risk factors associated with elder abuse by adult children. Health care providers should improve detection of elder abuse and support at-risk caregivers. Policy makers may consider cultural sensitive approaches to address elder abuse.

## Introduction

Elder abuse, also referred to as elder mistreatment or elder maltreatment, is an important public health and human rights issue. The prevalence of elder abuse ranges from 2.2% to 61.1% around the world [1]. The 2008 US National Elder Mistreatment Study suggests that more than 10% of the community-dwelling aging population reported elder abuse or potential neglect in the past year [2]. Existing literature has shown that elder abuse may result in psychological distress, increased morbidity and mortality in older adults [3,4]. In addition, elder abuse is linked to increased health services utilization, such as nursing home placement, emergency department utilization and hospitalization [5–9].

Elder abuse in family settings is an important public health issue that demands more attention. About 43.5 million of adult family caregivers provide care for people aged 50 years and over in the US [10]. A national study based on 1996 datasets suggested that an estimated 551,000 older adults in family settings were abused or neglected, and approximately 90% of perpetrators were family members [11]. Family caregiving is a potential ground that might increase the tendencies for elder abuse, given the role of caregiving burden and stress in elder abuse [12]. Prior research indicated that common perpetrators for elder abuse in family settings include spouse and adult children. However, given women may live longer than men with an average of seven years, there may be more and more adult children caring for their aging mothers, as compared to spouse caregivers [13]. Based on a report by the National Center on Elder Abuse (NCEA) in 1998, adult children are the most frequent perpetrators of the older adults. In addition, based on findings in a Chinese community in Hong Kong, Yan et al. [14] suggested that the majority of elder abuse cases (75%) were committed by older adults’ adult children. Therefore, it is necessary to investigate the elder abuse from the perspective of adult children.

Nevertheless, very few studies have examined the elder abuse reported by adult children, and the existing limited studies more focused on family caregivers as a whole [15,16]. Earlier findings showed a prevalence of 12%–55% for elder abuse reported by family caregivers [17]. More recent studies in Hong Kong, the prevalence was 42.3% and 62.3% for the abusive behaviors toward the older adults with dementia reported by family caregivers [18,19]. In the UK, 52% family caregivers reported some abusive behavior and 34% reported abusive behaviors happening “at least sometimes” in the past three months [20]. In Japan, 30% of participants reported some kind of abuse of older adults with clinically mild cognitive impairment [21]. These studies have helped us to understand elder abuse by family caregivers across diverse populations, but we have limited knowledge regarding elder abuse by adult children in the U.S Chinese population [22].

The demographic growth of U.S. Chinese older adults warrants a deeper understanding of their health and aging issue [23]. The Chinese community is the largest and the fastest growing Asian American subgroup populations in the United States, with an estimated number of 4 million [24]. Compared with the 15% population growth rate among U.S. older adults, the population of U.S. Chinese adults aged 65 and above has increased by 55% in the past decade [25]. At the same time, the U.S. Chinese population is older in average age and less acculturated among U.S. immigrant groups [26]. It is reported that more than 80% of Chinese older adults in the U.S. are foreign born [27]. With the Chinese families immigrating to the U.S., the younger generations who are more acculturated may be less likely to adhere to traditional cultural values and practices [28]. In contrast, a prior study indicated that the U.S. Chinese older adults still have high expectations for adult children to take the caregiving obligation [29]. This growing discrepancy in cultural values may result in family conflicts and increased caregiver burden [28].

In part due to the linguistic and cultural barriers, as well as social isolation, the U.S. Chinese older adults are more likely to be dependent on family caregivers, which may predispose them to greater risk of elder abuse, based on the Social Exchange Theory that “elder abuse is the result of the elder’s increasing dependence on the caregiver” [12]. However, in order to maintain family harmony and honor, the Chinese older adults may be reluctant to disclose their abused experiences and may tend to deny the abusive situation to preserve face values [30]. Therefore, reported by the older adults may underestimate the nature and extent of elder abuse in the family settings among the U.S. Chinese community. To assess the prevalence of elder abuse by adult children is an alternative approach in understanding this topic.

To expand our current knowledge on elder abuse with regards to comprehensive estimate on the prevalence of elder abuse by adult children among Chinese older adults, this study aims to 1) investigate the prevalence of elder abuse reported by Chinese adult children in a community-dwelling population in the greater Chicago area, 2) examine the correlations between socio-demographic characteristics and elder abuse reported by Chinese adult children.

## Methods

### Population and settings

The present study is a cross sectional study of Chinese adult children in the greater Chicago area, with at least one living parent (father, mother or both). The project was initiated by synergistic community-academic collaboration among Rush Institute for Healthy Aging, Northwestern University, and many community-based social services agencies and organizations throughout the greater Chicago area. All study procedures were approved by the Institutional Review Boards of Rush University Medical Center.

In order to ensure study relevance to the well-being of the Chinese community and enhance participation, the present study implemented culturally and linguistically appropriate community recruitment strategies strictly guided by a community-based participatory research (CBPR) approach [31,32]. The community-academic partnership enables us to develop appropriate research methodology in accordance with Chinese cultural context, in which a community advisory board (CAB) plays a crucial role in providing insights and strategies for research conduct and sustaining community partnerships [33,34]. Board members include community stakeholders and residents enlisted through over twenty civic, health, social and advocacy groups, community centers and clinics in the city and suburbs of Chicago.

### Study design and procedure

The research team recruited the adult children from community centers, and also through the local advertisement in the greater Chicago area. Inclusion criteria were as follows: (1) aged 21 years and over(2)reside in the greater Chicago area (3) at least one parent is Chinese aged 60 years and older. The adult children who met the inclusion criteria were invited to participate in this study. The adult children who were younger than 21 years old or resided outside of the greater Chicago area, or both of whose Chinese parents were younger than 60 years old/deceased, were excluded from this study. Before the interviews, all participants gave the written informed consent.

For ensuring cultural and linguistic sensitivity, trained interviewers were recruited through community partners and were equipped with multicultural and multilingual abilities. They conducted face-to-face home interviews to collect data with participants in their preferred language and dialects, such as English, Mandarin, Cantonese, Taishanese or Teochew dialects. Before field interviews, all hired interviewers received an intensive training that included appropriate data collection techniques, survey questionnaire administration, in-person communication skills, basic understanding of health sciences research and mock-interview role play. During the data collection period, booster trainings combined with staff meeting were conducted one to two times a month to fortify specific aspects of in-person training, and additional trainings on new issues arose from the field work were provided as well [35].

## Measurements

### Socio-demographics

Basic demographic information was collected, including age (in years), gender, education level, annual income (in USD), and marital status, number of children, living arrangement, and country of origin, language preference and abilities. Education level was assessed by asking participants the years of the highest educational level completed, with a range of 0 to 17 years or more. Self-reported annual income was from all sources (wages, salaries, social security or retirement benefits, help from relatives, rent from property, etc.) and was categorized into seven groups: 1) $0–$4,999 per year 2)$5,000–$9,999 per year 3)$10,000–$14,999 per year 4)$15,000–$19,999 per year 5)$20,000–$24,999 per year 6)$25,000–$29,999 per year 7) Over $30,000 per year. Living arrangement was assessed by asking participants how many people live in their household except themselves. Country of origin was divided into Mainland China, Hong Kong/Macau, Taiwan, or others. The language was assessed by the preference and ability to speak English, Cantonese, Mandarin or Toisanese.

### Overall health status, quality of life, and health changes over the last year

Overall health status was measured by “In general, would you say your health is____” on a four-point scale (1 = poor, 2 = fair, 3 = good, 4 = very good). Quality of life was assessed by “The quality of my life is____” also on a four-point scale (1 = poor, 2 = fair, 3 = good, 4 = very good). Health change in last year was measured by asking “Compared to one year ago, how would you rate your health now?” on a five-point scale (1 = much worse; 2 = somewhat worse; 3 = about the same; 4 = somewhat better; and 5 = much better than one year ago). Health changes were then categorized into three groups: (1) improved health; (2) same health; and (3) worsened health.

### Elder abuse reported by adult children

We assessed the elder abuse reported by adult children by using Caregiver Abuse Screen (CASE). The CASE is a brief screening tool with dichotomous (yes/no) response categories, and is used for detecting elder abuse, without enquiring directly about the specific abusive behaviors [36]. Participants were asked: 1) Do you sometimes have trouble making your parents control his/her temper or aggression? 2) Do you often feel you are being forced to act out of character or do things you feel bad about, because of your parents? 3) Do you find it difficult to manage your parents’ behavior? 4) Do you sometimes feel that you are forced to be rough with your parents? 5) Do you sometimes feel you can’t do what is really necessary or what should be done for your parents? 6) Do you often feel you have to reject or ignore for your parents? 7) Do you often feel so tired and exhausted that you cannot meet your parents’ needs? 8) Do you often feel you have to yell at your parents? We also add two items to evaluate financial abuse: 9) Do you have access to your parents’ bank account, checks, credit cards, and investment accounts? 10) Do you sometimes feel it is your responsibility to conduct financial transactions on your parent’s behalf, for what you think is their best interest? A “yes” response to any questions indicated the risk of exercising abuse.

The original scale was demonstrated to have acceptable to good internal consistency (Cronbach’s α = 0.75) [37]. A Chinese version of the CASE showed the similar reliability coefficient (Cronbach’s α = 0.77), and its test-retest Spearman correlation coefficient was 0.54 [38]. Additionally, the CASE scores correlated with the previously substantiated instruments of Indicator of Abuse (IOA) (r = 0.41, p < 0.001) and the Hwalek-Sengstock Elder Abuse Screening Test (HSEAST) (r = 0.26; p < 0.025) [39]. The Chinese version of the CASE in this study was forward and backward translated by bilingual and bicultural researchers, and the principal investigator who is bilingual and bicultural cross-examined the translation along with the CAB members to ensure validity. The ten-item scale in this study sample has demonstrated a moderate internal consistency (Cronbach’s α = 0.74) (range from 0 to 10).

### Data analysis

Descriptive statistics was used to summarize socio-demographic characteristics and the elder abuse reported by adult children. Chi-squared tests were used to compare the bivariate socio-demographic differences between the “Yes” to Any Caregiver Abuse Screen Item group and “No” to Any Caregiver Abuse Screen Item group. The Pearson Correlation coefficients were calculated to examine the correlations between socio-demographic/socioeconomic variables and any elder abuse reported by adult children. All statistical analyses were conducted using SAS, Version9.2 (SAS Institute Inc., Cary, NC).

## Results

### Sample characteristics

Among 548 participants in the study, 65.5% were women, 81.5% were married, and 27.1% had and an annual income below $10,000. The mean age of the participants was 47.6 (SD = 10.4, Range 22.4–75.7). The majority (90%) of the participants was born in Mainland China, more than half of them live in the U.S for more than 10 years, and 67.7% were interviewed in Cantonese as their preferred dialect. Elder abuse was found in over half (59.8%) of the participants, as shown in table 1. Compared with the group reported “No” to any Caregiver Abuse Screen item, the group reported Yes” had a greater proportion of adult children aged under 40 years old (29.8% vs. 20.7%, p < .05), had an educational level of more than 13 years (42.4% vs. 21%, p < .001), had an annual income of at least $15,000 (60.9% vs. 41.5%, p < .001), with no children (18.4% vs. 9.1%, p < .01), not born in Mainland China (13.2% vs. 5.5%, p < .05), had lived more than 11 years in the U.S. (67.7% vs. 59.2%, p < .05), and were able to speak English (32% vs. 17.9%, p < .001).

### Prevalence of elder abuse reported by adult children

Prevalence of elder abuse reported by adult children is presented in table 2. Endorsement of each Caregiver Abuse Screen item ranged from 4.8% to 25.1%. Having trouble making parents control his/her temper or aggression was the most prevalent item among adult children (25.1%), followed by it is children’s responsibility to conduct financial transactions on parent’s behalf (22.8%) and cannot do what is necessary for parents (22.5%). Feeling being forced to be rough with parents was least reported (4.8%). If the item 1, 3, 5, 9, 10 were removed separately, the prevalence of elder abuse would varied between 55.0% and 58.6%.

### Correlation between different items of elder abuse

The correlation between different items of elder abuse reported by adult children is shown in table 3. Both “act out of character because of parents “and “reject or ignore for parents” were significantly correlated with all other Caregiver Abuse Screen items. The correlation coefficient (r) of all significant correlations varied between 0.10 and 0.49. “Conduct financial transactions on parent’s behalf” was correlated to “reject or ignore for parents” at r = 0.10, p < 0.05, while was correlated to “have access to parents’ financial account” at r = 0.49, p < 0.001.

### Correlation between socio-demographics and elder abuse

The correlation between socio-demographics and elder abuse reported by adult children is presented in table 4. Elder abuse was significantly correlated with younger age (r = −0.10, p < .05), higher level of education (r = 0.20, p < .001), higher income (r = 0.14, p < .01), more years in the U.S. (r = 0.12, p < .05), not born in Mainland China (r = −0.13, p < .01), and English-speaking (r = 0.16, p < .001).

## Discussion

To our knowledge, this is the first study examining elder abuse by adult children among U.S Chinese families. Our study found that 59.8% of participants self-disclosed the risk of having committed abuse towards their older parents; adult children were more likely to report elder abuse if they were younger, with higher level of education, higher income, living in the U.S. for more years, not born in Mainland China, and was able to speak English.

It may be challenging to systematically compare the prevalence of elder abuse in this study with existing studies conducted with family caregivers due to multiple reasons. First, the majority of studies not only included adult children, but also spouses, children-in-law, grandchildren, and other relatives. Second, most studies focused on caregivers who were taking caring of older adults with dementia or disabilities. Third, the measurements that were commonly used differed from this study. In a Hong Kong study sampled 122 family caregivers with 62.3% adult children and 76.2% women involved, 62.3% of them acknowledged that they displayed abusive behaviors toward care recipients in the past month [19]; Different from our current study, Yan et al.’sstudy utilized the Revised Conflict Tactic Scales (CTS2)as the instrument to measure elder abuse, and the recipients were older adults with dementia.

To compare the results reported by adult children in this study with those observed in the U.S. Chinese aging population [28,40,41], this study displayed a much higher prevalence rate. In a study sampled 3,159 community-dwelling Chinese older adults aged 60 and over in Chicago, the prevalence of the overall elder abuse was 15% when using the Hwalek-Sengstok Elder Abuse Screening Test (H-S/EAST) and the Vulnerability to Abuse Screening (VASS) [28]. The lower rate of elder abuse reported by the U.S. Chinese older adults might be due to the sense of shame and cultural stigma on elder abuse [33,42]; while the adult children, under the impact of acculturation, might perceive less cultural barrier to report elder abuse. Moreover, the way the items of the CASE were phrased, such as “being forced”, “finds it difficult” “so tired and exhausted that cannot”, did not criticize adult children implicitly and might make them feel more comfortable to provide the real responses.

Most current studies on elder abuse are cross-sectional studies, and the underlying mechanism remains unclear [43]. It is possible that the high prevalence of elder abuse is due to the changing values and the caregiving burden that the adult children are having. Depending on the years living in the U.S., the adult children have become acculturated to Western culture’s emphasis on individualism. Compared with their older parents, the adult children are likely to have a different perspective on filial piety [44,45], that is, children being respectful, obedient, and obligated to provide support and care for older parents both emotionally and financially [46]. Take the younger generation in Hong Kong as an example, “love and care” has been perceived as paying for parents’ institutional care [47]. A recent study indicated that more than half of the Chinese older adults placed high expectations on filial piety [29]. This discrepancy between the expectations of filial piety and the receipt of filial care may aggravate family conflicts and elder abuse [48]. Apart from the changing values, the caregiving burden is a significant factor associated with elder abuse by family caregivers [19,49,50]. Many adult children are undertaking multiple responsibilities, including career, household duties, and children to take care. Therefore, it is highly possible that the adult children are experiencing a great deal of stress and burden when caring for the older parents [51]. Based on the Situational theory, the stressed and overburdened adult children may become abusive toward their vulnerable parents if they are not able to manage those caregiving demands [12]. Future studies are needed to verify these hypotheses.

Regarding the relationship between socio-demographic factors and elder abuse reported by adult children, this study demonstrated that younger age and higher educational level were positively correlated with elder abuse. Previous studies yielded mixed findings. In a study of 92 caregivers to community-dwelling Chinese older adults in Taiwan, participants with younger age (r = −0.315, p < .01) and higher levels of education (r = 0.219, p < .05) were more likely to perform psychologically abusive behavior [49]. However, a majority of studies did not find the correlation between caregivers’ age and abusive behaviors toward older adults with dementia or cognitive impairment [18,21,52,53]. Similarly, the caregiver’s educational level was also not related to verbal or physical elder abuse, as shown in Yan et al. [19]’s study. In our current study, it is possible that younger adult children have less experience and insufficient preparation for the heavy caregiving demands; In addition, adult children with higher level of education, also correlated to higher income (r = 0.51, p < .001), are more likely to live in suburban area and have fewer family members to share the caregiving burden with them. These conditions may lead to higher possibility of elder abuse. Currently, there is little previous literature to corroborate above hypothesis. Future research should be conducted to clarify the association between the socio-demographics of caregivers and elder abuse, and explore the influencing mechanism.

The results of this study should be interpreted with limitations. First, the generalizability of our findings to national or international Chinese populations is unwarranted given the location specificity and inner-ethnic variation. Second, the nature of its cross-sectional study design and correlation analysis makes it difficult to postulate on the temporal relationships between socio-demographic variables and elder abuse reported by adult children. Third, the measurements for elder abuse are based on a subjective assessment with a yes/no format, which does not reflect the severity and frequency of elder abuse by adult children. The items for financial abuse may overestimate elder abuse, considering the particular linguistic barriers that the U.S. Chinese older adults are facing. Nevertheless, it may still underestimate participants’ actual elder abuse owing to social desirability. Future studies are needed to develop a more culturally sensitive instrument to screen elder abuse by adult children, and can add a multiple-point scale to capture the occurrence rate of elder abuse. Additionally, longitudinal study is necessary to provide further information on the correlates found in the current study.

Despite these limitations, this study has important implications for researchers, health care providers and policy makers. Firstly, it calls for more investigation on elder abuse in U.S. Chinese community from the perspective of perpetrators, including the adult children. Further efforts are needed to understand the risk factors associated with elder abuse reported by adult children. Secondly, health care providers should improve detection of elder abuse in the community settings. Screening elder abuse by family caregivers, such as adult children, is an effective alternative approach to detect elder abuse, considering the Chinese older adults have the tendency to underreport or conceal their abused experiences due to cultural values. Screening elder abuse put health care provider into a proactive position, and is the first step for developing future interventions to prevent elder abuse [54]. It is important for health providers to consider elder abuse interventions from a familial and interpersonal perspective. To understand the dynamics of elder abuse within the context of family caregiving will help health providers to develop a more comprehensive intervention program. Previous research proved that educational intervention and support for at-risk caregivers are effective in mitigating abusive behaviors [55–57]. Thirdly, the findings from this study also have implications to the Elder Justice Act of 2010, which is the first federal legislation addressing elder abuse at the national level and has been implemented currently [58]. The policy maker should place more attention on elder abuse by adult children, and consider relevant cultural issues [59–61].

## Conclusion

The elder abuse by adult children is a pervasive health and public issue among U.S. Chinese population. For adult children, younger age, higher levels of education, higher income, living in the U.S. for more years, not born in Mainland China, and English-speaking are positively correlated with elder abuse against U.S. Chinese older adults. Longitudinal research is needed to advance our knowledge on the risk factors associated with elder abuse by adult children.

## Figures and Tables

**Table 1:** Characteristics of study participants by reporting results of caregiver abuse screen

	Yes to Any Caregiver Abuse Screen item (N = 326)	No to Any Caregiver Abuse Screen item (N = 219)	X^2^	d.f.	P value
**Age, N (%)**					
20–29	14 (4.3)	1 (0.5)			
30–39	83(25.5)	44(20.2)			
40–49	108(33.2)	82(37.6)			
50–59	75(23.1)	58(26.6)			
60–69	41(12.6)	32(14.7)			
70–79	4(1.2)	1(0.5)	11.2	5	< .05
**Gender**					
Male	120(36.8)	69(31.5)			
Female	206(63.2)	150(68.5)	1.6	1	0.2
**Education (years), N (%)**					
0–6	19(5.8)	28(12.8)			
7–12	169(51.8)	145(66.2)			
13–16	100(30.7)	33(15.1)			
17+	38(11.7)	13(5.9)	29.7	3	< .001
**Income (USD), N (%)**					
$0–$4,999	36(11.1)	27(12.4)			
$5,000–$9,999	41(12.6)	43(19.8)			
$10,000–$14,999	50(15.4)	57(26.3)			
$15,000–$19,999	58(17.9)	24(11.1)			
$20,000–$24,999	36(11.1)	23(10.6)			
$25,000–$29,999	26(8.0)	8(3.7)			
Over $30,000	78(24.0)	35(16.1)	24.1	6	< .001
**Marital Status, N (%)**					
Married	260(79.8)	185(84.5)			
Separated	4(1.2)	1(0.5)			
Divorced	16(4.9)	12(5.5)			
Widowed	7(2.2)	7(3.2)			
Never Married	39(12.0)	14(6.4)	6	4	0.2
**Living Arrangement, N (%)**					
Living alone	8(2.5)	7(3.2)			
1	48(14.7)	26(11.9)			
2–3	159(48.8)	95(43.4)			
4 or more	111(34.1)	91(41.6)	3.9	3	0.28
**Number of Children, N (%)**					
0	60(18.4)	20(9.1)			
1–2	216(66.3)	157(71.7)			
3 and more	50(15.3)	42(19.2)	9.4	2	< .01
**Years in the U.S., N (%)**					
0–10	95 (30.2)	89 (40.8)			
11–20	106 (33.7)	66 (30.3)			
21–30	78 (24.8)	49 (22.5)			
31+	36 (11.4)	14 (6.4)	8.4	3	< .05
**Country of Origin, N (%)**					
Mainland China	283 (86.8)	207 (94.5)			
Hong Kong/Macau	22 (6.8)	8 (3.7)			
Taiwan	5 (1.5)	0 (0.0)			
Other	16 (4.9)	4 (1.8)	9.9	3	< .05
**Language Preference, N (%)**					
Cantonese	224 (68.7)	144 (65.8)			
Mandarin	46 (14.1)	12(5.5)			
Toisanese	30 (9.2)	54 (24.7)			
English	26 (8.0)	9 (4.1)	32.7	3	< .001
**Language Ability-Cantonese, N(%)**					
Yes	273 (83.7)	198 (90.4)			
No	53 (16.3)	21 (9.6)	5	1	< .05
**Language Ability-English, N(%)**					
Yes	104 (31.9)	40 (18.3)			
No	222(68.1)	179 (81.7)	13.4	1	< .001
**Language Ability-Mandarin, N(%)**					
Yes	125 (38.3)	70 (32.0)			
No	201 (61.7)	149 (68.0)	2.3	1	0.13
**Language Ability-Toisanese, N(%)**					
Yes	133 (40.8)	139 (63.47)			
No	193 (59.2)	80 (36.53)	26.9	1	< .001
**Overall Health Status, N (%)**					
Very good	34 (10.6)	12 (5.6)			
Good	162 (50.5)	111 (51.9)			
Fair	107 (33.3)	81 (37.9)			
Poor	18 (5.6)	10 (4.7)	4.7	3	0.19
**Quality of Life, N (%)**					
Very good	9 (2.8)	6 (2.8)			
Good	121 (37.7)	71 (33.2)			
Fair	161 (50.2)	125 (58.4)			
Poor	30 (9.4)	12 (5.6)	4.7	3	0.2
**Health Changes Over the Last Year, N (%)**					
Improved	24 (7.5)	11 (5.1)			
Same	212 (66.0)	144 (67.3)			
Worsened	85 (26.5)	59 (27.6)	1.2	2	0.56

**Table 2:** Prevalence of elder abuse reported by adult children

Caregiver Abuse Screen Items	Yes, N (%)	No, N (%)
1. Do you sometimes have trouble making your parents control his/her temper or aggression?	137 (25.1)	409 (74.9)
2. Do you often feel you are being forced to act out of character or do things you feel bad about, because of your parents?	40 (7.3)	505 (92.3)
3. Do you find it difficult to manage your parents’ behavior?	104 (19.1)	442 (81.0)
4. Do you sometimes feel that you are forced to be rough with your parents?	26 (4.8)	520 (95.2)
5. Do you sometimes feel you can’t do what is really necessary or what should be done for your parents?	123 (22.5)	423 (77.5)
6. Do you often feel you have to reject or ignore for your parents?	59 (10.8)	487 (89.2)
7. Do you often feel so tired and exhausted that you cannot meet your parents’ needs?	69 (12.7)	477 (87.3)
8. Do you often feel you have to yell at your parents?	93 (17.0)	453 (83.0)
9. Do you have access to your parents’ bank account, checks, credit cards, and investment accounts?	107 (19.6)	439 (80.4)
10. Do you sometimes feel it is your responsibility to conduct financial transactions on your parent’s behalf, for what you think is their best interest?	124 (22.8)	421 (77.3)

**Table 3:** Correlations between different items of elder abuse reported by adult children

	Trouble control parents’ temper	Act out of Character	Difficult to manage parents’ behavior	Rough with parents	Cannot do what is necessary	Reject or ignore	Cannot meet needs	Yell at parents	Access to parents’ Bank	Financial transactions
Trouble control parents’ temper	1									
Act out of Character	0.28#	1								
Difficult to manage parents’ behavior	0.48#	0.39#	1							
Rough with parents	0.23#	0.40#	0.29#	1						
Cannot do what is necessary	0.30#	0.27#	0.37#	0.17#	1					
Reject or ignore	0.14#	0.27#	0.27#	0.15#	0.26#	1				
Cannot meet needs	0.32#	0.25#	0.28#	0.17#	0.25#	0.41#	1			
Yell at parents	0.30#	0.36#	0.38#	0.36#	0.30#	0.26#	0.27#	1		
Access to parents’ Bank	0.06	0.16#	0.07	0.17#	−0.01	0.11+	0.12+	0.11*	1	
Financial transactions	0.11*	0.12+	0.07	0.08	0	0.10*	0.07	0.05	0.49#	1

* p<.05,

+ p<.01,

# p<.001

**Table 4:** Correlations between socio-demographic characteristics and elder abuse reported by adult children

	Age	Gender	Edu	Income	MS	Living	Child Num	Yrs. in US	China born	LP-CT	LP-ENG	LP-Mand	LP-Toi	LA-CT	LA-ENG	LA-Mand	LA-Toi	OHS	QOL	HC	EA
Age	1																				
Gender	0.03	1																			
Edu	−0.34#	−0.02	1																		
Income	−0.22#	0.01	0.51#	1																	
MS	0.12+	−0.03	−0.04	0.02	1																
Living	−0.19#	−0.01	−0.06	−0.01	0.25#	1															
ChildNum	0.28#	0.03	−0.23#	−0.06	0.36#	0.47#	1														
Yrs. in US	0.35#	0.01	0.10 *	0.17#	−0.10*	−0.15#	0.17#	1													
Chinaborn	0	0.03	−0.19#	−0.08	0.20#	0.19#	0.12+	−0.35#	1												
LP-CT	−0.02	−0.06	−0.24#	−0.12+	0.03	0.09*	0.06	−0.05	0.16#	1											
LP-ENG	−0.12+	−0.05	0.27#	0.13 +	−0.19#	−0.21#	−0.17#	0.40 #	−0.53#	−0.38#	1										
LP-Mand	−0.03	0.06	0.46#	0.28 #	0.01	−0.01	−0.11+	−0.10*	0.04	−0.50#	−0.09*	1									
LP-Toi	0.13 +	0.06	−0.27#	−0.17#	0.08 *	0.03	0.13+	−0.07	0.13 +	−0.62#	−0.11+	−0.15#	1								
LA-CT	0.01	−0.04	−0.40#	−0.27#	−0.01	0	0.10*	0.06	0.03	0.49#	−0.05	−0.78#	0.07	1							
LA-ENG	−0.22#	0	0.45#	0.42 #	−0.16#	−0.12+	−0.16#	0.30#	−0.33#	−0.16#	0.44#	0.13+	−0.21#	−0.15#	1						
LA-Mand	−0.13+	0.01	0.34#	0.22 #	0	0.02	−0.12+	−0.08	0.10*	−0.04	−0.15#	0.44#	−0.22#	−0.38#	0.23#	1					
LA-Toi	−0.05	0.03	−0.39#	−0.27#	0.09*	0.20#	0.18#	−0.21#	0.27#	−0.03	−0.17#	−0.32#	−0.43#	0.23#	−0.19#	−0.19#	1				
OHS	−0.21#	−0.01	0.20#	0.17#	0.02	0.10*	0	−0.08	−0.04	−0.12+	0.07	0.09*	0.02	−0.13+	0.15#	0.04	0.02	1			
QOL	0.07	−0.04	0.03	−0.06	−0.02	−0.10*	−0.04	0.06	−0.10*	−0.09*	0.13+	−0.05	0.06	0.06	0	−0.06	−0.09*	−0.09*	1		
HC	−0.15#	−0.08	0.11+	0.04	−0.01	0.04	−0.04	0	−0.06	−0.02	0.02	−0.01	0.02	−0.02	0.11+	0.02	0.02	0.27#	−0.12+	1	
EA	−0.10*	−0.05	0.20#	0.14+	−0.06	−0.07	−0.08	0.12*	−0.13+	0.03	0.08	0.14+	−0.21#	−0.10*	0.16#	0.07	−0.22#	0.05	0.01	0.02	1

* p<.05,

+ p<.01,

# p<.001.

**Notes:** Edu: Education, MS: Marital Status, Living: Living Arrangement, Yrs. in U.S. : Years in the US, ChildNum: Number of children, LP-CT: Language preference_ Cantonese,

LP-ENG: Language preference_ English, LP-Mand: Language preference_ Mandarin, LP-Toi: Language preference_ Toisanese,

LA-CT: Language ability-Cantonese, LA-ENG: Language ability-English, LA-Mand: Language ability-Mandarin,

LA-Toi: Language ability-Toisanese, OHS: Overall Health Status, QOL: Quality of Life, HC: Health Changes over the Last Year, EA: Elder Abuse.
